# Clinical Significance of Ascitic Fluid Polymorphonuclear Leukocyte Percentage in Patients With Cirrhosis Without Spontaneous Bacterial Peritonitis

**DOI:** 10.14309/ctg.0000000000000614

**Published:** 2023-07-12

**Authors:** Lillian Dawit, Vivian Lee, David Lehoang, Cameron Furey, Aneesa Chowdhury, Thu Anne Mai, Varun Angajala, Joo Hye Park, Kevork Khadarian, Rosemary She, Maria Vergara-Lluri, Jeffrey Kahn, Jennifer L. Dodge, Takeshi Saito

**Affiliations:** 1 Division of Gastrointestinal and Liver Diseases, Department of Medicine, Keck School of Medicine, University of Southern California, Los Angeles, California, USA;; 2 Department of Pathology, Keck School of Medicine, University of Southern California, Los Angeles, California, USA;; 3 USC Research Center for Liver Diseases, Keck School of Medicine, University of Southern California, Los Angeles, California, USA;; 4 Department of Population and Public Health Sciences, Keck School of Medicine, University of Southern California, Los Angeles, California, USA.

**Keywords:** polymorphonuclear leukocyte count, polymorphonuclear leukocyte percentage, spontaneous bacterial peritonitis, cirrhosis, URM/URIM, Hispanic/Latinx

## Abstract

**INTRODUCTION:**

Absolute polymorphonuclear leukocyte (PMN) count (PMN-C) ≥250 cells/mm^3^ in ascites is the diagnostic hallmark of spontaneous bacterial peritonitis (SBP) and is associated with high morbidity and mortality. However, the clinical significance of ascitic PMN percentage (PMN-%) and PMN-C in the absence of SBP as additional biomarkers for mortality and future incidence of SBP has not been determined.

**METHODS:**

This retrospective cohort included adults with cirrhosis undergoing first-recorded paracentesis with initial PMN-C < 250 cells/mm^3^ at 2 tertiary medical centers between 2015 and 2020. Patients with prior SBP were excluded. Outcomes were death and SBP development. Cox regression estimated hazard ratios (HRs) for risk of death and SBP development and Akaike information criterion to compare model fit.

**RESULTS:**

Three hundred eighty-four adults (73% male, median age 58 years, 67% with alcohol-associated cirrhosis, median PMN-C 14 cells/mm^3^ [interquartile range 5–34], and median PMN-% 10% [interquartile range 4–20]) were included in this study. Univariate risk of death increased 10% per 25-unit increase in PMN-C (95% confidence interval 1.01–1.21, *P* = 0.03) and 19% per 10-unit increase in PMN-% (95% confidence interval 1.06–1.33, *P* = 0.003) with PMN-% demonstrating better model fit in assessing mortality risk (Akaike information criterion: 1,044 vs 1,048, respectively). In models adjusted for age, chronic hepatitis C virus infection, and Model for End-Stage Liver Disease-Sodium, PMN-% was associated with risk of death (PMN-% 10%–29%, HR 1.17, *P* = 0.50; PMN-% ≥ 30% group, HR 1.94, *P* = 0.03; vs PMN-% < 10%) and SBP development (PMN-% 10%–29%, HR 1.68, *P* = 0.07; PMN-% ≥ 30%, HR 3.48, *P* < 0.001; vs PMN-% < 10%).

**DISCUSSION:**

Our results suggest PMN-% at first paracentesis represents a better biomarker compared with PMN-C for assessing risk of death and future SBP development in patients with PMN-C < 250 cells/mm^3^.

## INTRODUCTION

The high morbidity and mortality of cirrhosis, the 11th leading cause of death worldwide (1), are largely driven by the development of severe complications such as spontaneous bacterial peritonitis (SBP), variceal hemorrhage, acute kidney injury, and liver cancer. SBP, a bacterial infection of ascites, is the most common infectious disease in patients with advanced cirrhosis, occurring in up to 30% of hospitalized cases (2–4). The onset of SBP frequently results in acute kidney injury, septic shock, and ultimately multiorgan failure (5,6). Accordingly, the mortality rate of SBP is high, estimated at approximately 20%–50% (3,7–10), and could be as high as 80% if not promptly diagnosed and managed with effective antimicrobials (3,11). Hence, the identification of individuals at high risk of developing SBP is a crucial milestone in mitigating the disease burden of advanced cirrhosis. Prior studies have identified risk factors of SBP development, such as low ascitic fluid protein, high degree of liver failure, circulatory dysfunction, and low platelet counts (12,13). In addition, elevated C-reactive protein level and high Model for End-Stage Liver Disease–Sodium (MELD-Na) score have been used as markers that predict SBP-related mortality (11,14,15). While these biomarkers greatly aid the management of patients with cirrhosis, the prevalence of SBP remains high worldwide (16), indicating the necessity for better prognostic markers.

The pathophysiology of SBP is considered multifactorial, mediated through intestinal bacterial overgrowth and impaired antibacterial immunity in the gastrointestinal tract, leading to bacterial translocation, colonization, and infection into the peritoneal cavity (17,18). The sensing of bacterial pathogens by peritoneal macrophages triggers the production of inflammatory cytokines necessary for the recruitment of polymorphonuclear leukocytes (PMNs) into the peritoneum. Therefore, the presence of PMNs themselves may reflect the degree of bacterial translocation into the peritoneal cavity (19). In fact, in healthy individuals, there exists <20 mL of free intraperitoneal fluid, containing fewer than 300 cells/mm^3^, mostly macrophages and lymphocytes, with PMN count (PMN-C) accounting for fewer than 10 cells/mm^3^ (20,21).

Currently, the diagnosis of SBP is made through the detection of PMN-C ≥ 250 cells/mm^3^ in the ascites and culture yielding a microorganism in the absence of a surgically treatable source of infection (22–26). These empiric SBP diagnostic criteria have been established from prior studies showing that the greatest sensitivity was reached with a cutoff of 250 cells/mm^3^, although the greatest specificity was reached with a cutoff of 500 PMNs/mm^3^ (27–31). It is conceivable that the degree of bacterial translocation correlates with increasing PMN-C and the proportion of PMNs (hereafter, PMN percentage [PMN-%]) over other cell types even in those who do not meet the diagnostic criteria of SBP (PMN-C < 250 cells/mm^3^). However, the clinical significance of PMN-C and PMN-% as biomarkers for future incidence of SBP and associated mortality in patients without SBP has never been explored. In this study, we aim to elucidate the utility of PMN-C and PMN-% as biomarkers to identify high-risk populations for the occurrence of SBP and mortality.

## METHODS

### Study design and population

This is a retrospective study of adults aged 18 years or older with ascites due to cirrhosis who underwent first standard-of-care paracentesis with initial PMN-C < 250 cells/mm^3^ at 2 tertiary care centers in Los Angeles, California, Los Angeles County + University of Southern California Medical Center and Keck Hospital of University of Southern California, between 2015 and 2020. The diagnosis of cirrhosis was determined based on the combination of clinical, laboratory, histopathological, and radiographic data. All paracenteses were performed in both asymptomatic and symptomatic patients for diagnostic and therapeutic purposes in the emergency department, inpatient, and outpatient settings. Indications for paracenteses included the following: (i) new-onset ascites, (ii) clinical suspicion for SBP (including symptoms such as fever and abdominal pain or new complications of portal hypertension such as onset of hepatic encephalopathy or renal impairment), (iii) symptomatic alleviation for large-volume ascites, and (iv) the presence of ascites in hospitalized cirrhosis patients. Staff physicians in the emergency medicine, internal medicine, and radiology departments performed the bedside paracenteses. Those who had a history of SBP, noncirrhotic ascites, or recent exposure to antibiotics were excluded. Approval from the Institutional Review Board was obtained (#HS-17-00883), and written informed consent was waived, given the retrospective nature of this research. All research was performed in accordance with relevant guidelines and regulations.

### Laboratory and clinical data

Demographical, clinical, and laboratory data during the first-recorded paracentesis were collected from the electronic medical records. This included age, sex, ethnicity, etiology of cirrhosis, date of paracentesis, and serum and ascitic fluid laboratory data. Specifically, laboratory values such as ascitic fluid total protein and values used to calculate the serum ascites albumin gradient and MELD-Na were collected. Four study subjects had serum ascites albumin gradient < 1.1; however, each had laboratory, clinical, and imaging findings consistent with the diagnosis of cirrhosis with evidence of portal hypertension and no alternate explanation for ascitic fluid development. Child Pugh scores were not collected for analysis because of the subjective nature of the scoring system such as degree of encephalopathy and size of ascites, which can be variable in description depending on the provider (32). Subsequent clinical outcomes were recorded including future diagnosis of SBP, major intra-abdominal procedures, and death or discharge to hospice.

### Outcomes

The primary outcome was death including those dying from any cause or discharged to hospice. The secondary outcome was SBP development defined as a diagnosis of SBP with ascitic fluid PMN-C ≥ 250 cells/mm^3^. Patients were followed up from initial paracentesis to the outcome of interest with patients censored on the date of liver transplant, transjugular intrahepatic portosystemic shunt placement, transfer to outside facility, or last known follow-up with the hospital; thus, all patients contributed to the analysis up to the time of censoring.

### Statistical analysis

Characteristics at initial paracentesis were summarized as medians (interquartile ranges [IQRs]) and frequencies (percentages). The Kaplan-Meier method with the log-rank test was used to estimate the rates of death and SBP development with 95% confidence intervals (CIs) and identify differences in event rates. The FINDCUT macro was used to identify potential cut points within PMN-% and PMN-C associated with death based on the Contal and O'Quigley technique, Cox hazard ratio (HR) maximization, and *P*-value minimization. Candidate cut points were tested in univariable Cox proportional hazards regression to assess for best model fit, indicated by the lowest Akaike information criterion (AIC) values, and confirmed using the C-statistic. Optimal PMN-% cutoffs were included in a series of multivariable Cox regression models to evaluate the association between death and PMN-% cutoffs adjusted for (i) baseline factors with univariable significance level < 0.1 and (ii) baseline factors and PMN-C. Due to missing MELD-Na, primary models were adjusted for serum creatinine with a sensitivity analysis among those with available MELD-Na. The PMN-% cutoffs were also tested for association with SBP development. Collinearity was assessed and variance inflation factors were confirmed to be less than 10. Sensitivity and specificity with 95% CIs were calculated for the PMN-% cutoff of 30% and PMN-C cutoff of 115 cells/mm^3^ and compared using an exact test of proportions. In an exploratory analysis, death rates for PMN-% cutoffs were compared with patients with PMN-C ≥ 250 cells/mm^3^ excluded from the primary cohort; although the latter are treated patients with SBP, this analysis provides perspective on the risk associated with PMN-% relative to PMN-C ≥ 250 cells/mm^3^. Statistical analyses were executed using SAS version 9.4 (SAS Institute Inc, Cary, NC).

## RESULTS

### Patient characteristics

A total of 384 adults (73% male, 78% Hispanic, median age 58 years [IQR 51–63]) who underwent first paracentesis were included in the study. Baseline characteristics during first paracentesis are summarized in Table 1. Cirrhosis etiology was predominantly alcohol-associated liver disease (ALD). The median MELD-Na was 20 (IQR 14–25) with no detectable difference in MELD-Na score noted between both tertiary care medical centers. A total of 54 patients had PMN-% > 30 (median PMN-C 41, IQR 36–51) and 186 patients had PMN-% < 10. Forty-three of 384 (11.2%) patients ultimately underwent liver transplant, transjugular intrahepatic portosystemic shunt, or other intra-abdominal surgery and were censored during these events.

**Table 1.** Patient demographics and baseline characteristics of serum and ascitic fluid during first paracentesis in the overall primary cohort of patients with PMN-C < 250 cells/mm^3^

	Total (N = 384)
Male, n (%)	281 (73)
Age, yr, median (IQR)	58 (51–63)
Hispanic, n(%)	301 (78)
Etiology (ALD), n (%)	257 (67)
Serum creatinine, mg/dL, median (IQR)	0.90 (0.66–1.42)
HCV, n (%)	77 (20)
INR, median (IQR)	1.55 (1.31–1.95)
Total bilirubin, mg/dL, median (IQR)	2.50 (1.20–5.70)
Serum albumin, g/dL, median (IQR)	2.70 (2.30–3.20)
Serum sodium, mmol/L, median (IQR)	136 (132–139)
Ascitic fluid total protein, g/dL, median (IQR)	1.30 (0.90–1.80)
MELD-Na, median (IQR)	20 (14–25)
PMN-C of first paracentesis fluid, median (IQR)	14 (5–34)
PMN-% of first paracentesis fluid, % (IQR)	10 (4–20)
Paracentesis to future SBP development, mo (IQR)	4.27 (1.05–14.73)
Paracentesis to death or last follow-up, mo (IQR)	6.00 (1.33–20.98)

Analysis included those with available serum creatinine (N = 377), INR (N = 349), total bilirubin (N = 371), serum albumin (N = 370), serum sodium (N = 376), ascitic fluid total protein (N = 150), and MELD-Na (N = 346).

ALD, alcohol-associated liver disease; HCV, chronic hepatitis C virus infection; INR, international normalized ratio; IQR, interquartile range; MELD-Na, Model for End-Stage Liver Disease–Sodium; PMN-%, polymorphonuclear leukocyte percentage; PMN-C, polymorphonuclear leukocyte count; SBP, spontaneous bacterial peritonitis.

### Risk of death by PMN-C

**Figure 1.**
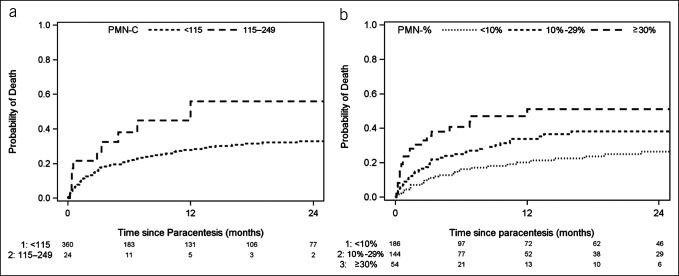
The cumulative incidence of death in cirrhosis patients without SBP following the first paracentesis. The Kaplan-Meier analysis of mortality by PMN-C (**a**) or PNM-% (**b**) at the time of the initial paracentesis. PMN-%, polymorphonuclear leukocyte percentage; PMN-C, polymorphonuclear leukocyte count (cells/mm^3^); SBP, spontaneous bacterial peritonitis.

In the primary cohort of patients with PMN-C < 250 cells/mm^3^, 101 patients died during follow-up, of which 25% (N = 25) were discharged to hospice. Risk of death increased by 10% per 25-unit increase in PMN-C (HR 1.10, 95% CI 1.01–1.21, *P* = 0.03) (Table 2). A potential PMN-C cutoff of 115 cells/mm^3^ was identified, corresponding to 6-month cumulative incidences of death of 22% (95% CI 17–27) for PMN-C < 115 cells/mm^3^ and 38% (95% CI 21–63, *P* = 0.03) for 115–249 cells/mm^3^ (Figure 1a). AIC was 1,048 for both the continuous form and for a cutoff of 115 cells/mm^3^ (Table 2). Risk of death was increased for patients with PMN-C 115–249 cells/mm^3^ (HR 2.06, *P* = 0.03) compared with that for patients with PMN-C < 115 cells/mm^3^.

**Table 2.** Univariable HRs for risk of death and SBP development associated with PMN count and percent with AIC values to assess best model fit

PMN at paracentesis	Risk of death				Risk of SBP development			
	HR (95% CI)	*P*	AIC	C-statistic	HR (95% CI)	*P*	AIC	C-statistic
PMN-C (cells/mm^3^)								
Per 25 cells/mm^3^ increase	1.10 (1.01–1.21)	0.03	1,048	0.585	1.14 (1.03–1.25)	0.01	756	0.630
≥115 (vs <115)	2.06 (1.07–3.97)	0.03	1,048	0.527	2.33 (1.11–4.86)	0.02	757	0.533
PMN-%								
Per 10% increase	1.19 (1.06–1.33)	0.003	1,044	0.609	1.29 (1.14–1.45)	<0.001	747	0.655
3 categories:			1,039	0.618			745	0.665
<10%	1.00				1.00			
10%–29%	1.67 (1.07–2.60)	0.02			1.91 (1.13–3.22)	0.02		
≥30%	2.90 (1.93–4.72)	<0.001			4.11 (2.20–7.66)	<0.001		

AIC, Akaike information criterion; CI, confidence interval; HR, hazard ratio; PMN, polymorphonuclear leukocyte; PMN-%, PMN percentage; PMN-C, PMN count; SBP, spontaneous bacterial peritonitis.

### Risk of death by PMN-% versus PMN-C

Risk of death was assessed by PMN-% versus PMN-C. Risk of death increased by 19% per 10-unit increase in PMN-% (HR 1.19, 95% CI 1.06–1.33, *P* = 0.003) with PMN-% demonstrating better model fit over PMN-C per 25-unit increase in assessing mortality risk (AIC 1,044 vs 1,048, respectively) (Table 2). Potential cutoffs were identified for PMN-% (10% and 30%) with AIC values for PMN-% being lower than for PMN-C (Table 2). PMN-% categories of <10%, 10%–29%, and ≥30% had the lowest AIC of all potential cutoffs (1,039) and the highest C-statistic (0.618) with risk of death increasing 1.67-fold (95% CI 1.07–2.60, *P* = 0.02) for PMN-% 10–29 and 2.90-fold (95% CI 1.93–4.72, *P* < 0.001) for PMN-% ≥ 30 compared to PMN-% < 10. The 6-month cumulative incidence of death increased from 16% for PMN-% < 10 (95% CI 11–23) to 25% for PMN-% 10–29 (95% CI 18–34) and 41% for PMN-% ≥ 30 (95% CI 28–57) (Figure 1b). In multivariable analysis, PMN-%, but not PMN-C 115–249 cells/mm^3^, remained significantly associated with risk of death (Table 3). Specifically, for PMN-% ≥ 30 compared with PMN-% < 10, risk of death remained elevated (HR 2.91, *P* < 0.001) after adjusting for characteristics at paracentesis (age, creatinine, and chronic hepatitis C virus infection) and was only slightly attenuated (HR 2.67, *P* = 0.004) after adjusting for PMN-C 115–249 cells/mm^3^ and after adjusting for MELD-Na (HR 1.94, *P* = 0.03).

**Table 3.** Univariable and multivariable Cox proportional hazards regression models for the association of risk of death with PMN percent and count (cells/mm^3^) among those with PMN < 250 cells/mm^3^ at first paracentesis

HR (95% CI), *P*				
		Multivariable^a^		
Characteristic at paracentesis	Univariable	Adjusted for baseline characteristics	Adjusted for PMN-C ≥ 115 cells/mm^3^	Adjusted for MELD-Na^b^
PMN-%				
<10%	1.00	1.00	1.00	1.00
10%–29%	1.67 (1.07–2.60), 0.02	1.50 (0.96–2.35), 0.07	1.50 (0.96–2.33), 0.07	1.17 (0.74–1.84), 0.50
≥30%	2.90 (1.93–4.72), <0.001	2.91 (1.68–5.06), <0.001	2.67 (1.37–5.19), 0.004	1.94 (1.08–3.48), 0.03
Age (per year)	1.03 (1.01–1.05), 0.01	1.03 (1.01–1.05), 0.01	1.03 (1.01–1.05), 0.008	1.05 (1.02–1.07), <0.001
Creatinine (per 1 mg/dL)	1.15 (1.03–1.28), 0.01	1.17 (1.05–1.31), 0.005	1.17 (1.05–1.31), 0.005	—
HCV (vs non-HCV)	2.24 (1.47–3.42), <0.001	1.12 (1.38–3.26), 0.001	2.14 (1.39–3.28), <0.001	2.21 (1.42–3.43), <0.001
PMN-C ≥ 115 (vs <115 cells/mm^3^)	2.06 (1.07–3.97), 0.03	—	1.23 (0.54–2.82), 0.62	—
MELD-Na (per 1 unit)	1.08 (1.05–1.11), <0.001	—	—	1.10 (1.07–1.14), <0.001

CI, confidence interval; HCV, chronic hepatitis C virus infection; HR, hazard ratio; MELD-Na, Model for End-Stage Liver Disease–Sodium; PMN, polymorphonuclear leukocyte; PMN-%, PMN percentage; PMN-C, PMN count.

a Multivariable models adjusted for age, serum creatinine, and HCV at paracentesis; N = 377 due to missing serum creatinine values.

b Sensitivity analysis included those with available MELD-Na (N = 346) adjusted for age, HCV, and MELD-Na at paracentesis.

The sensitivity and specificity were also evaluated for the cutoffs of PMN-% ≥ 30, but <250 cells/mm^3^, and PMN-C 115–249 cells/mm^3^ (Table 4). The sensitivity of PMN-% ≥30 was higher than that for PMN-C 115–249 cells/mm^3^ in assessing death (20.8% vs 9.9%, *P* = 0.002). However, the specificity was lower with PMN-% ≥ 30 compared with that with PMN-C 115–249 cells/mm^3^ (death: 88.3% vs 95.0%, *P* < 0.001).

**Table 4.** Sensitivity and specificity for PMN-% vs PMN-C in assessing death and SBP development among those with PMN < 250 cells/mm^3^ at first paracentesis

	Death			SBP development		
	PMN-% ≥ 30%	PMN-C ≥ 115 cells/mm^3^	*P*	PMN-% ≥ 30%	PMN-C ≥ 115 cells/mm^3^	*P*
Sensitivity	20.8 (13.4–30.0)	9.9 (4.8–17.5)	0.002	23.0 (13.4–32.6)	10.8 (3.7–17.9)	0.001
Specificity	88.3 (84.0–91.8)	95.0 (91.8–97.3)	<0.001	88.1 (84.5–91.7)	94.8 (92.4–97.3)	<0.001

PMN, polymorphonuclear leukocyte; PMN-%, PMN percentage; PMN-C, PMN count; SBP, spontaneous bacterial peritonitis.

### Risk of SBP development by PMN-% versus PMN-C

In the cohort, 74 patients subsequently developed SBP. In univariate analysis, risk of SBP development increased by 29% per 10-unit increase in PMN-% (HR 1.29, 95% CI 1.14–1.45, *P* < 0.001) and 14% per 25-unit increase in PMN-C (HR 1.14, 95% CI 1.03–1.25, *P* = 0.01) with PMN-% demonstrating better model fit in assessing future SBP development risk (AIC 747 and 756, respectively) (Table 2). PMN-% of 10–29 and ≥30 had HRs of 1.91 (95% CI 1.13–3.22, *P* = 0.02) and 4.11 (95% CI 2.20–7.66, *P* < 0.001), respectively, for risk of SBP development compared with PMN-% < 10 and had a lower AIC (745) than PMN-% per 10-unit increase (747) and all forms of PMN-C (Table 2). The 6-month cumulative incidence of SBP development increased from 7% for PMN-% < 10 (95% CI 4–12) to 22% for PMN-% 10–29 (95% CI 15–31, *P* = 0.04) and 32% for PMN-% ≥ 30 (95% CI 20–50, *P* < 0.001).

In multivariable analysis, increased risk of SBP development remained significantly associated with PMN-% across all adjusted models (Table 5). Notably, there were significant increases in risk of SBP development for the PMN-% 10–29 group (HR 1.86, *P* = 0.02) and the PMN-% ≥ 30 group (HR 3.86, *P* < 0.001) when compared with PMN-% < 10 independent of creatinine and PMN-C. In addition, when adjusting for MELD-Na, increased risk of SBP development remained higher in the PMN-% 10–29 group (HR 1.68, *P* = 0.07) and the PMN-% ≥ 30 group (HR 3.48, *P* < 0.001) as compared with PMN-% < 10, with the latter achieving statistical significance. When accounting for PMN-%, no significant difference in risk of SBP development was detected for PMN-C 115–249 cells/mm^3^ vs PMN-C < 115 cells/mm^3^ (HR 0.98, *P* = 0.97).

**Table 5.** Univariable and multivariable Cox proportional hazards regression models for the association of spontaneous bacterial peritonitis development risk with PMN percent and count, among those with PMN < 250 cells/mm^3^ at first paracentesis

HR (95% CI), *P*				
		Multivariable^a^		
Characteristic at paracentesis	Univariable	Adjusted for baseline characteristics	Adjusted for PMN-C ≥ 115 cells/mm^3^	Adjusted for MELD-Na^b^
PMN-%				
<10%	1.00	1.00	1.00	1.00
10%–29%	1.91 (1.13–3.22), 0.02	1.86 (1.10–3.14), 0.02	1.86 (1.10–3.14), 0.02	1.68 (0.97–2.91), 0.07
≥30%	4.11 (2.20–7.66), <0.001	3.83 (2.06–7.13), <0.001	3.86 (1.88–7.92), <0.001	3.48 (1.80–6.72), <0.001
Creatinine (per 1 mg/dL)	0.78 (0.57–1.06), 0.11	0.78 (0.57–1.07), 0.13	0.78 (0.57–1.07), 0.13	—
PMN-C ≥ 115 (vs <115 cells/mm^3^)	2.33 (1.11–4.86), 0.02	—	0.98 (0.41–2.36), 0.97	—
MELD-Na (per 1 unit)	1.03 (0.99–1.06), 0.10	—	—	1.02 (0.99–1.06), 0.18

CI, confidence interval; HR, hazard ratio; MELD-Na, Model for End-Stage Liver Disease-Sodium; PMN, polymorphonuclear leukocyte; PMN-%, PMN percentage; PMN-C, PMN count.

a Multivariable models adjusted for serum creatinine at paracentesis; N = 377 due to missing serum creatinine values.

b Adjusted for MELD-Na at paracentesis; N = 346 due to missing MELD-Na values.

In the analysis of sensitivity and specificity, the sensitivity of PMN-% ≥ 30, but <250 cells/mm^3^, was higher than for PMN-C 115–249 cells/mm^3^ in assessing SBP development (23.0% vs 10.8%, *P* = 0.001), but the specificity of PMN-% ≥ 30, but <250 cells/mm^3^, was lower than that of PMN-C 115–249 cells/mm^3^ (88.1% vs 94.8%, *P* < 0.001) (Table 4).

### Comparison With PMN-C ≥ 250 cells/mm^3^

In an exploratory analysis, we compared the mortality rates of individuals with PMN-C < 250 cells/mm^3^ with those of patients with PMN-C ≥ 250 cells/mm^3^ who were excluded from the primary cohort (N = 42, 71% male, median age 58 years) (see Supplemental Table 1, Supplemental Digital Content 1, http://links.lww.com/CTG/A975, which summarizes patient demographic and baseline laboratory values in the overall population stratified by PMN-C < 250 cells/mm^3^ and PMN-C ≥ 250 cells/mm^3^). No significant differences in death rates at 6 months were detected for PMN ≥ 250 cells/mm^3^ compared with PMN-% ≥ 30 (46% vs 41%, *P* = 1.00) or PMN-C 115–249 cells/mm^3^ (46% vs 38%, *P* = 0.35). Cumulative incidences of death rates increased from 22% for PMN-C < 115 cells/mm^3^ (95% CI 17–27) to 38% for PMN-C 115–249 cells/mm^3^ (95% CI 21–63, *P* < 0.01) and 46% for PMN ≥ 250 cells/mm^3^ (95% CI 32–63, *P* < 0.01) (see Supplemental Figure 1, Supplemental Digital Content 2, http://links.lww.com/CTG/A976, which illustrates cumulative incidence of death after first paracentesis plotted by PMN-C [1A] and PMN-% [1B]). In a multivariable analysis, PMN-%, but not PMN ≥ 250 cells/mm^3^, remained significantly associated with risk of death (see Supplemental Table 2, Supplemental Digital Content 3, http://links.lww.com/CTG/A977, which summarizes univariable and multivariable regression models for the association of risk of death with PMN percent and count). Specifically, for PMN-% ≥ 30, but <250 cells/mm^3^, compared with PMN-% < 10, risk of death remained elevated (HR 2.96, *P* < 0.001) after adjusting for characteristics at paracentesis (age, creatinine, and chronic hepatitis C virus infection) and was only slightly attenuated (HR 2.87, *P* < 0.001) after adjusting for PMN-C ≥ 250 cells/mm^3^ and after adjusting for MELD-Na (HR 1.85, *P* = 0.04).

## DISCUSSION

This retrospective cohort study characterized the clinical significance of leukocyte count and composition of cirrhotic ascites at first standard-of-care paracentesis with the goal of defining new biomarkers that aid in identifying individuals at high risk of developing SBP and associated mortality. Our study found both PMN-C and PMN-% are significantly associated with rates of mortality and SBP development. These associations held true in analyses of both continuous value measurements and categorized data. These findings suggest that the increasing number and proportion of PMNs may reflect a greater degree of bacterial translocation into the ascitic fluid whether it is above or below the current diagnostic threshold for SBP of PMN ≥ 250 cells/mm^3^.

Notably, our study revealed that PMN-% is a better predictor of mortality and SBP development than PMN-C. We additionally found that PMN-% more than doubled the sensitivity of assessing these outcomes than PMN-C at the expense of a 6.7% decrease in specificity. This increased sensitivity importantly identifies at-risk patients who likely require closer monitoring and immediate management to reduce the complications of SBP and risk of mortality. While our study is the first to propose the superiority of PMN-% over PMN-C as a biomarker in assessing the future occurrence of SBP, previous studies demonstrated the significance of PMN-% in cases with SBP. One study (n = 64) reported that there exists an association between PMN-% and mortality, and PMN-% serves as an independent predictive factor for short-term or in-hospital mortality (33). Another study with 28 cases of SBP also suggested that patients with PMN-% > 85% have a significantly increased mortality (34). Given the better model fit of PMN-% over PMN-C for assessing risk of mortality and SBP development, our study suggests the inclusion of PMN-% in the diagnosis and management of SBP to identify high-risk individuals and improve clinical outcomes in this population.

Considering the possible explanation for the superiority of PMN-% over PMN-C as a biomarker for bacterial translocation into the peritoneum, we postulate that the influence of medical interventions for ascites volume control serves as a significant factor. Because PMN-C represents the cell population density, it is significantly influenced by the volume of ascitic fluid; therefore, standard-of-care nonprocedural interventions for the management of ascites, such as fluid and salt restrictions, the use of diuretics, and albumin infusions could all affect this parameter. It is also important to note that the efficiency of PMN recruitment into the peritoneum could be altered by large-volume paracenteses or repeated therapeutic paracenteses for ascites volume reduction. This is because PMN infiltration into the peritoneum is largely facilitated by the inflammatory cytokines and chemokines produced by PMs, following the recognition of bacterial pathogen-associated molecular patterns (35). As such, the manipulation of ascitic fluid volume by therapeutic interventions greatly alters the local concentration of bacterial pathogen-associated molecular patterns and humoral factors facilitating the neutrophil chemotaxis into the peritoneum, thereby confounding the utility of PMN-C as the surrogate of bacterial translocation. By contrast, PMN-% is unlikely influenced by the volume of ascitic fluid or these medical interventions because it is normalized by other cell types but not with the ascites volume.

Regarding the clinical utility of PMN-C, our study revealed that patients with PMN-C 115–249 cells/mm^3^ had comparable mortality rates with those with SBP (PMN-C ≥ 250 cells/mm^3^), although the latter is noted to be a population who had been treated with standard-of-care antibiotics therapy. This finding is consistent with the outcomes of previous studies. One retrospective cohort analyzing SBP-naive patients (n = 272) at first paracentesis found that the incidence of SBP development was significantly higher in cases with PMN ≥ 100 cells/mm^3^ when compared with patients with PMN ≤ 100 cells/mm^3^, while all other clinical characteristics were similar among the 2 groups (36). This study concluded that PMN-C is an independent predictor of subsequent SBP development with a relative risk of 1.352 (1.162–1.499) per 50 cells/mm^3^ increase. Another study of 178 subjects stratified by PMN-C at index paracentesis (<125 cells/mm^3^, 125–250 cells/mm^3^, and >250 cells/mm^3^) also found that patients with PMN-C > 125 cells/mm^3^ are at high risk for 1-year mortality, with rates similar to those of patients with SBP (37). These findings, along with ours, suggest the necessity of lowering the diagnostic threshold of SBP or considering PMN-C ≥ 115 cells/mm^3^ as an indication for SBP prophylaxis, given the comparable death rates of patients with PMN-C 115–249 cells/ mm^3^ with those of patients with SBP (PMN-C ≥ 250 cells/mm^3^) who were treated with standard-of-care antibiotics therapy.

The major strength of our study is the large sample size collected from 2 independent tertiary medical centers with highly distinctive health-care systems: the combination of safety net and nonsafety net hospitals, which greatly broadens the diversity of our study population. Another strength is the comprehensiveness of the data collection on salient clinical and laboratory parameters, enabling the detailed analysis of first-time paracenteses in patients with cirrhosis with an adequate follow-up period to adverse outcomes and precise exclusion of patients with a history of SBP or recent antibiotic exposure, including antibiotic prophylaxis. These factors together allowed us to elucidate the utility of PMN-% as a biomarker that aids in assessing SBP development and mortality. The limitations associated with this study include the retrospective study design and the relatively fewer number of subjects with higher PMN-% (PMN-% ≥ 30, N = 54) than patients with lower PMN-% (PMN-% < 10, N = 186; PMN% 10–29, N = 144). In addition, a validation cohort was not available; thus, results must be confirmed in an external cohort. Lastly, the predominance of ALD as the etiology of cirrhosis might limit the generalizability of our study outcome. This is because, although it remains controversial (38), it has been reported that ALD has a higher risk for the development of SBP and worsened outcomes compared with other etiologies (39–42).

Our study demonstrates that PMN-% serves as a novel biomarker that aids in identifying patients at high risk of SBP development and mortality. In addition, our study suggests that PMN-% is a superior marker to PMN-C in assessing these outcomes. Our observations propose the necessity of reevaluating the threshold for the diagnosis of SBP to include PMN-% ≥ 30% or PMN-C ≥ 115 cells/mm^3^, regardless of symptoms or ascitic fluid culture positivity. Alternatively, we propose inclusion of these parameters in the indications for antibiotic prophylaxis to decrease near-future occurrences of SBP and mortality rates. To our knowledge, this study is the largest to date focusing on the clinical implications of PMN-C in patients without SBP and is the only study evaluating the prognostic utility of PMN-% in patients both with and without SBP. Follow-up studies with prospective study designs involving multiple medical centers with diverse patient populations are required to further validate our conclusions.

## CONFLICTS OF INTEREST

**Guarantor of the article:** Takeshi Saito, MD, PhD.

**Specific author contributions:** L.D.: leading conceptualization, data curation, investigation, methodology, writing—original draft, and writing—review and editing; supporting formal analysis, project administration. V.L.: coleading data curation, investigation, methodology, and writing—original draft; supporting conceptualization, writing—review and editing, and formal analysis. D.L.: supporting data curation, investigation. C.F.: supporting data curation. A.C.: supporting data curation. T.A.M.: supporting data curation. V.A.: supporting data curation. J.H.P.: supporting data curation. K.K.: supporting data curation. R.S.: supporting project administration. M.V.-L.: supporting project administration. J.K.: supporting project administration. J.L.D.: leading formal analysis; supporting investigation, data curation. T.S.: corresponding author; leading conceptualization, data curation, investigation, methodology, supervision, writing—original draft, writing—review and editing, project administration; supporting formal analysis.

**Financial support:** This study was supported in part by the USC Research Center for Liver Disease (P30DK048522).

**Potential competing interests:** None to report.

## Supplementary Material
