# Supplementary material for: Clinical Significance of Ascitic Fluid Polymorphonuclear Leukocyte Percentage in Patients With Cirrhosis Without Spontaneous Bacterial Peritonitis
Source: Clin Transl Gastroenterol. 2023 Jul 12;14(9):e00614. doi: 10.14309/ctg.0000000000000614 (PMC10522094; doi:10.14309/ctg.0000000000000614)
Supplement: Supplementary file 1 [file ct9-14-e00614-s001.pdf]

Supplemental Table 1, Supplemental Digital Content 1. Patient demographics and baseline characteristics of serum and ascitic fluid at time of first paracentesis in the overall cohort and stratified by PMN-C<250cells/mm^3 and PMN-C≥250cells/mm^3.

	Total(N=426)	PMN-C <250cells/mm^3 (N=384)	PMN-C ≥250cells/mm^3 (N=42)	P-value
Male (n,%)	311(73)	281(73)	30(71)	0.86
Age, years (median,IQR)	58(51-63)	58(51-63)	58(50-63)	0.86
Hispanic (n,%)	333(78)	301(78)	32(76)	0.74
Etiology (ALD,n,%)	280(66)	257(67)	23(55)	0.12
Serum Creatinine, mg/dL (median,IQR)	0.91(0.66-1.42)	0.90(0.66-1.42)	0.95(0.67-1.34)	0.62
HCV (n,%)	89(21)	77(20)	12(29)	0.20
INR (median,IQR)	1.55(1.32-2.00)	1.55(1.31-1.95)	1.68(1.35-2.24)	0.051
Total Bilirubin, mg/dL (median,IQR)	2.50(1.20-5.70)	2.50(1.20-5.70)	2.80(1.60-5.20)	0.46
Serum Albumin, g/dL (median,IQR)	2.70(2.30-3.20)	2.70(2.30-3.20)	2.65(2.20-3.10)	0.47
Serum Sodium, mmol/L (median,IQR)	136(132-139)	136(132-139)	138(132-140)	0.30
Ascitic Fluid Total Protein, g/dL (median,IQR)	1.30(0.90-1.80)	1.30(0.90-1.80)	1.40(0.90-2.05)	0.90
MELD-Na (median,IQR)	20(14-26)	20(14-25)	21(15-28)	0.20
PMN-C of First Paracentesis Fluid (median,IQR)	17(6-47)	14(5-34)	779(444-1501)	**<0.001**
PMN-% of First Paracentesis Fluid (%,IQR)	11(5-25)	10(4-20)	71(56-83)	**<0.001**
Follow-Up Time after Paracentesis (months,IQR)	5.7(1.2-19.6)	6.0(1.3-21.0)	1.1(0.5-12.1)	**0.04**

Key: IQR=interquartile range, ALD=Alcohol-associated liver disease, INR=International Normalized Ratio, MELD-Na=Model for End-Stage Liver Disease-Sodium
