# Supplementary material for: Clinical Significance of Ascitic Fluid Polymorphonuclear Leukocyte Percentage in Patients With Cirrhosis Without Spontaneous Bacterial Peritonitis
Source: Clin Transl Gastroenterol. 2023 Jul 12;14(9):e00614. doi: 10.14309/ctg.0000000000000614 (PMC10522094; doi:10.14309/ctg.0000000000000614)

Supplemental Figure 1, Supplemental Digital Content 2. The cumulative incidence of death after first paracentesis. 1A is plotted by PMN count (cells/mm^3). 1B is plotted by PMN percent with "≥30%" representing those with PMN%≥30 and <250cells/mm^3.

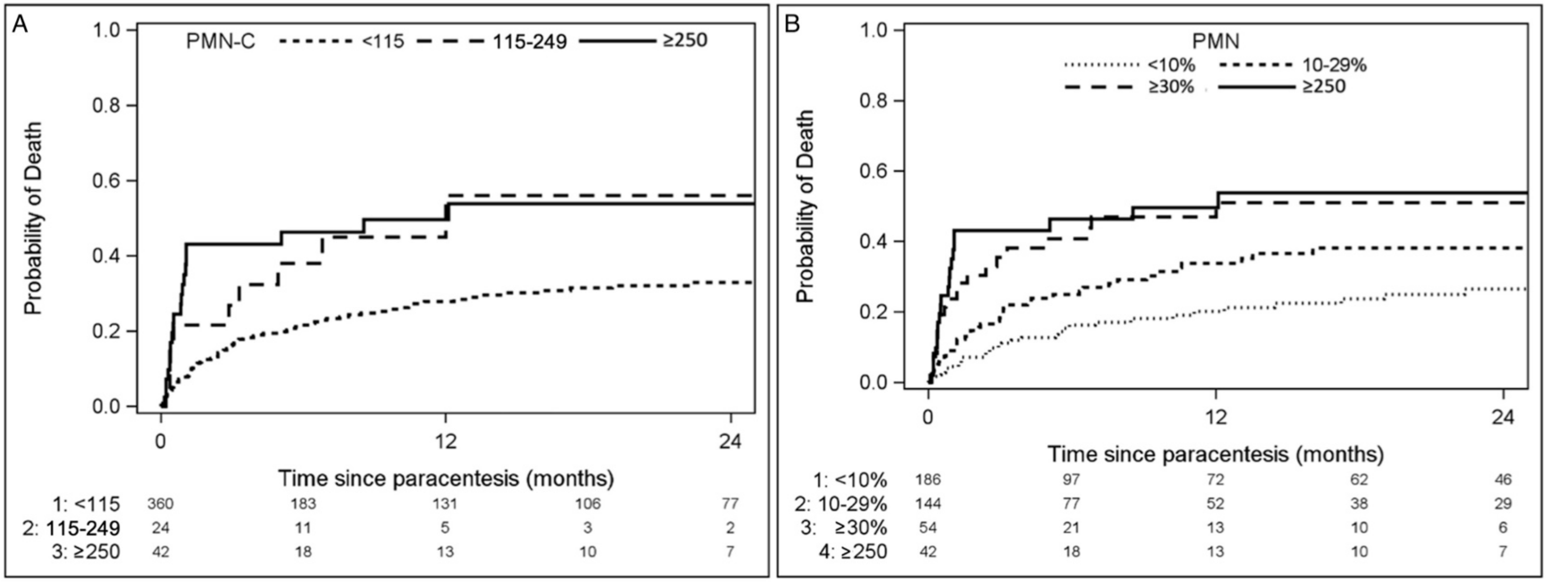

Supplement: Supplementary file 2 [file ct9-14-e00614-s002.pdf]
