# Supplementary material for: Clinical Significance of Ascitic Fluid Polymorphonuclear Leukocyte Percentage in Patients With Cirrhosis Without Spontaneous Bacterial Peritonitis
Source: Clin Transl Gastroenterol. 2023 Jul 12;14(9):e00614. doi: 10.14309/ctg.0000000000000614 (PMC10522094; doi:10.14309/ctg.0000000000000614)
Supplement: Supplementary file 3 [file ct9-14-e00614-s003.pdf]

Supplemental Table 2, Supplemental Digital Content 3. Univariable and multivariable Cox proportional hazards regression models for the association of risk of death with PMN percent and count (in cells/mm3).

Characteristic at paracentesis	Hazard Ratio (95% CI) p			
	Univariable	Multivariable*		
		Adjusted for baseline characteristics	Adjusted for PMN-C ≥250 cells/mm^3	Adjusted for MELD-Na^
PMN-%				
<10%	1.00	1.00	1.00	1.00
10-29%	1.65 (1.06-2.57) 0.03	1.50 (0.96-2.33) 0.07	1.50 (0.96-2.33) 0.07	1.15 (0.73-1.80) 0.55
≥30%	3.02 (1.93-4.72) <0.001	2.96 (1.88-4.66) <0.001	2.87 (1.66-4.96) <0.001	1.85 (1.04-3.30) 0.04
Age (per year)	1.02 (1.01-1.04) 0.01	1.02 (1.01-1.04) 0.01	1.02 (1.00-1.04) 0.02	1.04 (1.02-1.07) <0.001
Creatinine (per 1 mg/dL)	1.16 (1.05-1.28) 0.003	1.19 (1.08-1.32) <0.001	1.19 (1.08-1.32) <0.001	--
HCV (vs non-HCV)	2.07 (1.41-3.05) <0.001	1.93 (1.30-2.85) 0.001	1.93 (1.30-2.85) 0.001	2.33 (1.55-3.51) <0.001
PMN-C ≥250 (vs <250 cells/mm^3)	2.25 (1.39-3.63) 0.001	--	1.06 (0.57-1.96) 0.85	1.64 (0.84-3.22) 0.15
MELD-Na (per 1 unit)	1.09 (1.07-1.12) <0.001	--	--	1.12 (1.09-1.15) <0.001

*Multivariable models adjusted for age, serum creatinine, and HCV at paracentesis; N=419 due to missing serum creatinine values.
^A sensitivity analysis included those with available MELD-Na (N=384) adjusted for age, HCV, and MELD-Na at paracentesis.
